# Tuning cell surface charge in *E. coli* with conjugated oligoelectrolytes†
†Electronic supplementary information (ESI) available: Experimental details, additional figures. See DOI: 10.1039/c5sc03046c


**DOI:** 10.1039/c5sc03046c

**Published:** 2015-12-03

**Authors:** Chelsea Catania, Alexander W. Thomas, Guillermo C. Bazan

**Affiliations:** a Materials Department , University of California , Santa Barbara , CA 93106 , USA; b Center for Polymers and Organic Solids , Department of Chemistry and Biochemistry , University of California , Santa Barbara , CA 93106 , USA . Email: bazan@chem.ucsb.edu

## Abstract

Cationic conjugated oligoelectrolytes (COEs) varying in length and structural features are compared with respect to their association with *E. coli* and their effect on cell surface charge as determined by zeta potential measurements. Regardless of structural features, at high staining concentrations COEs with longer molecular dimensions associate less, but neutralize the negative surface charge of *E. coli* to a greater degree than shorter COEs.

## Abstract 2

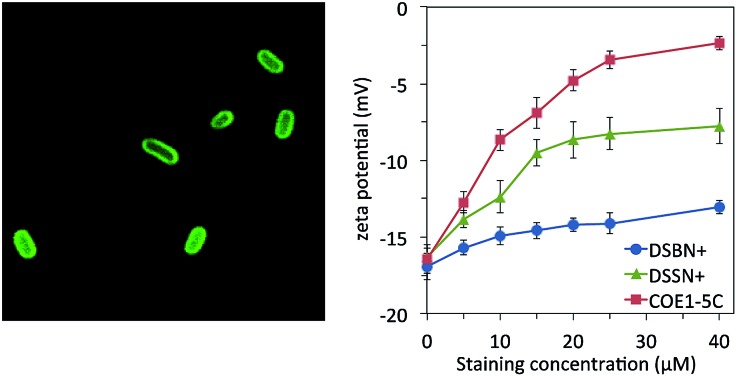
Conjugated oligoelectrolytes intercalate into and associate with membranes, thereby changing the surface charge of microbes, as determined by zeta potential measurements.

## Introduction

Although the manipulation of microbial cell properties offers the potential for harnessing and tuning the abilities of microorganisms, it remains a significant challenge due to the aqueous environment and overall structural complexity and diversity.^1^ Genetic engineering, while effective, is limited to materials the cell itself is capable of producing. Synthetic materials and molecular systems offer possible functionalities that are not encountered in nature. With this in mind, conjugated oligoelectrolytes (COEs) are synthetic molecules generally characterized by 3–5 π-conjugated repeat units (RUs) equipped with pendant ionic groups to impart solubility in polar media. COEs are related to conjugated polyelectrolytes used in optoelectronics,^2^–^5^ biosensing^1^,^6^–^8^ and bioimaging.^2^–^5^,^9^,^10^ COEs thus share attractive photophysical properties similar to those of their polymeric analogs, but have much smaller length scales, on par with biological architectures like proteins^11^,^12^ and lipid membranes.^13^–^17^ As such, a variety of COEs have found utility in bioimaging^18^–^24^ and biological detection schemes^25^–^30^ of their own.

**Chart 1:**
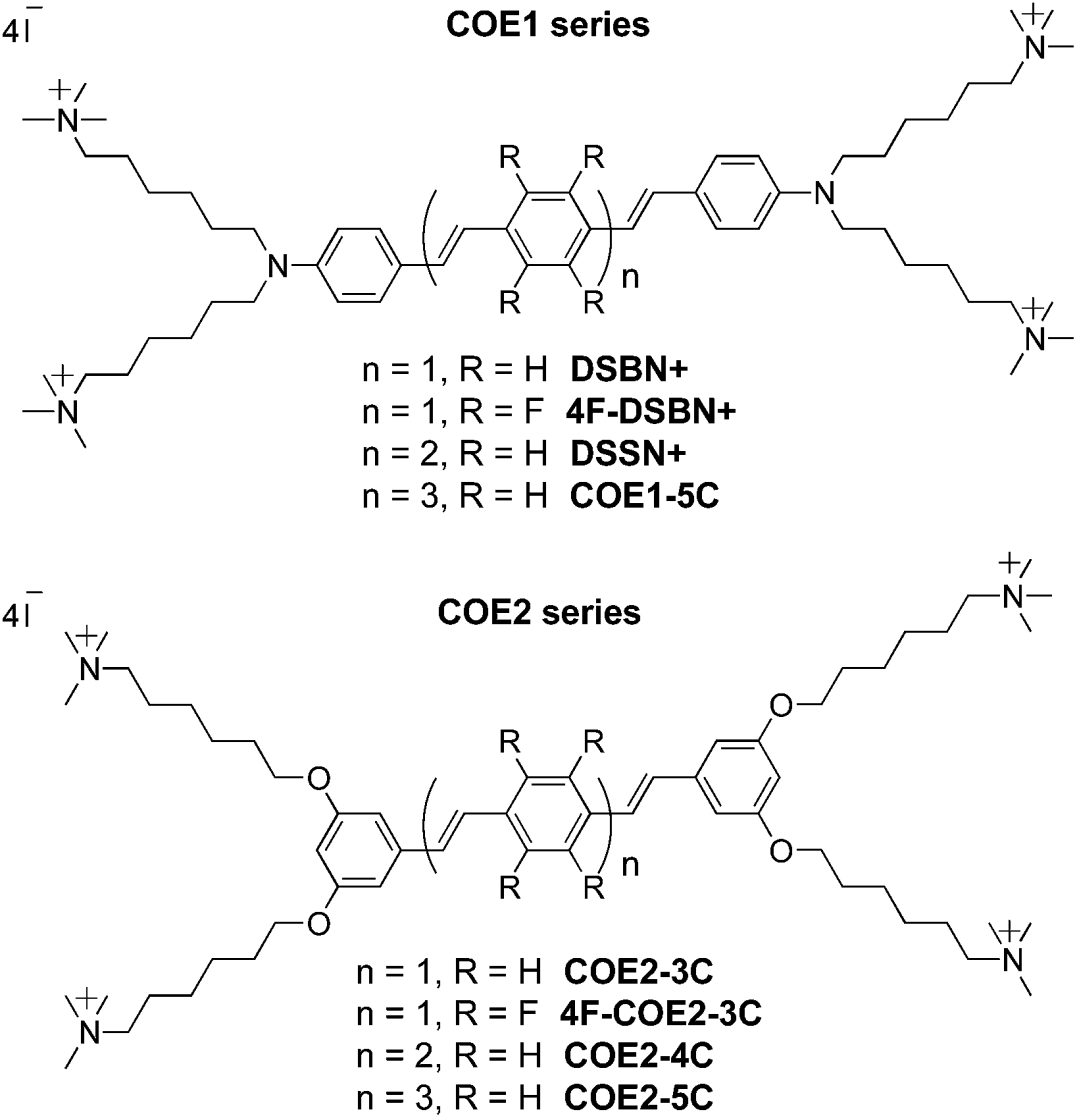
Chemical structures of COEs used in this study.

A distinct subset of COEs, and that used in this contribution, is distinguished by ionic functionalities tethered at the two terminal ends of a phenylenevinylene sequence. These bolaamphiphilic structures, in particular **DSBN+** and **DSSN+** (Chart 1), have been shown to spontaneously intercalate into lipid bilayers with a concomitant increase in fluorescence quantum yield.^14^ Polarized confocal microscopy has been used to demonstrate a preferential alignment of the COE's molecular long axis relative to the membrane plane. They have also been implicated in boosting the performance of a variety of microbial electronic devices^31^,^32^ employing organisms ranging from yeast,^14^ to *E. coli*^33^,^34^ and *Shewanella*,^35^,^36^ and even naturally occurring bacteria in wastewater.^37^ Although the exact mechanism of their action is still unclear,^36^,^38^,^39^ it is thought that the COEs' ability to intercalate into microbial membranes is paramount for linking intracellular metabolism to extracellular electrodes in these devices.^40^

While the lipid membrane intercalation of COEs is well-documented, other biological interactions of COEs and their consequences have not yet been studied. Previously we showed that an anionic COE analogous to **DSSN+** was prevented from incorporating into *E. coli* membranes most likely due to electrostatic repulsion from the innate negative surface charge of the cells.^40^ These negative charges occur mostly as ionized carboxyl and phosphate groups that are part of lipopolysaccharide (LPS) macromolecules composing the outer leaflet of most Gram-negative bacteria.^41^,^42^ Thus, electrostatic attraction between cationic COEs and these anionic sugars in the outermost extensions of *E. coli* are reasonable and should allow modulation of the overall surface charge of the cells.^43^–^45^ Furthermore, in studies concerning the effects of COEs on biological systems, COE concentrations are chosen in the low micromolar regime with no consideration given to the total number of cells; the amount of COE that associates with each cell and that which is left in solution remains to be quantified. With this purpose, we compare 8 COEs varying in molecular length and core substitutions for their association with *E. coli* and effect on cell zeta potential, finding a remarkable length dependence on these properties.

## Results and discussion

### Chemical structures

The chemical structures of the COEs used in this study are shown in Chart 1; their syntheses have been described in the literature.^14^,^34^,^46^ Their basic structure can be described by 3–5 phenylenevinylene repeat units (RUs) flanked on both ends by either an amine (COE1 series) or two *meta*-positioned alkoxy (COE2 series) linkages carrying trimethylammonium iodide terminated hexyl chains. Tetrafluorine substitution of the center phenylene ring of the 3-RU molecules offers variance of the central hydrophobic core to determine its role, if any, in cell association and cell surface charge.

### Confocal microscopy

**Fig. 1:**
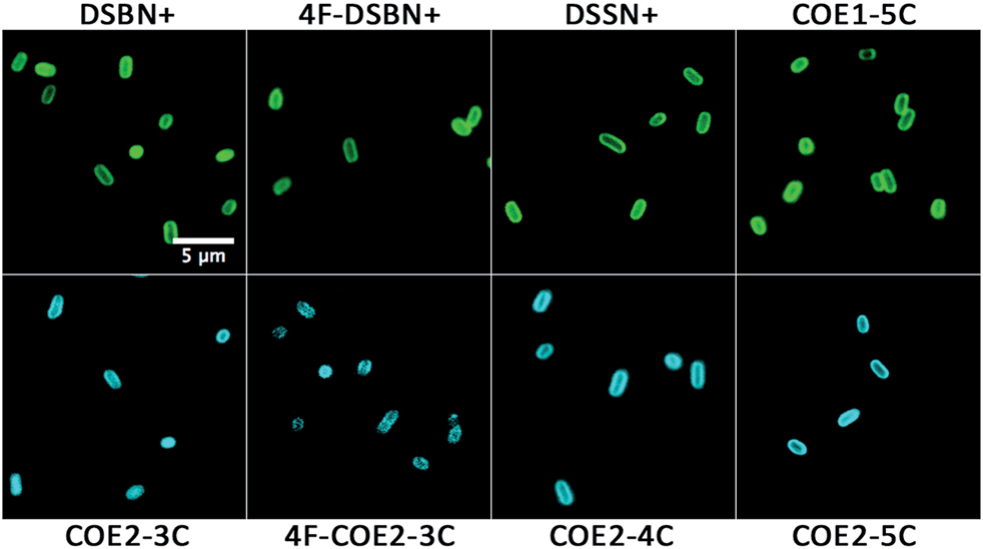
Laser scanning confocal micrographs of *E. coli* stained with 10 μM COE in PBS for 1 hour. Top row is COE1 series (green), bottom row is COE2 series (blue). Excitation wavelength was 405 nm for all images. 5 μm scale bar is the same for all images.

In order to first visualize how each COE interacts with *E. coli*, we exploited the photoluminescent π-conjugated core of the molecules for fluorescence microscopy. Cells were stained with 10 μM solutions of COE for 1 hour and imaged with a laser scanning confocal microscope, the results of which are shown in Fig. 1 (bright field images are shown in Fig. S1†). As anticipated based on the bolaamphiphilic structure shared by the molecules, all COEs display an emission pattern around the edges of cells consistent with membrane intercalation. In this regard, the substitution of alkoxy pendant linkages for amine or the addition of 4 fluorine atoms to the center phenyl ring of the 3-RU COEs provides no discernable difference in terms of observable cell localization in *E. coli*.

### Cell association studies

**Fig. 2:**
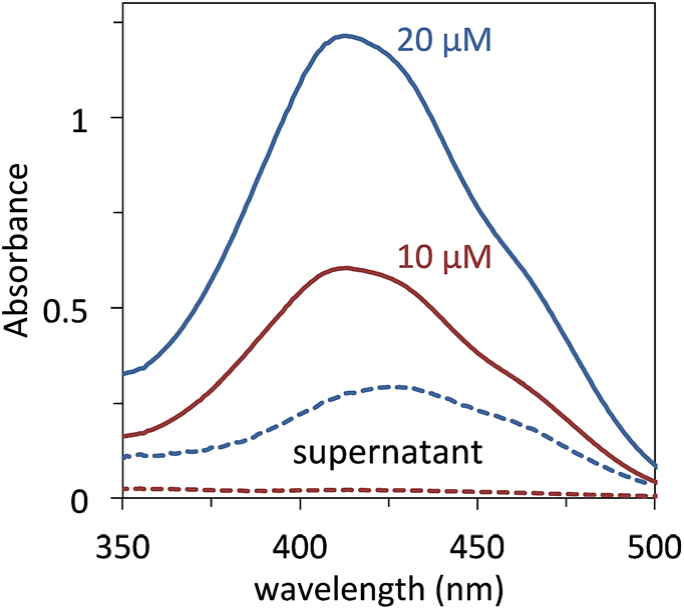
UV-vis absorption of 20 μM (blue, solid) and 10 μM (red, solid) **DSSN+** in PBS. After staining *E. coli* (OD_600 nm_ = 0.9) for 1 hour with these concentrations of **DSSN+**, the cells are centrifuged and the **DSSN+** remaining in the supernatant (dashed lines) is measured in order to determine how much COE associates with cells.

Taking advantage of the strong visible light absorbing properties provided by the conjugated core of the molecules,^14^ the amount of each COE that associates with *E. coli* in solution was quantified. Full experimental details are given in the Experimental section. Briefly, cells (OD_600 nm_ = 1.0) were stained in different concentrations of COE ranging from 1–40 μM for 1 hour in 50 mM phosphate buffered saline (PBS) solutions. All concentrations of COE used in this study were less than the critical aggregation concentration (CAC) reported for **DSBN+**, which is at 0.51 mM.^47^ A staining time of 1 hour was found sufficient to establish equilibrium within these experimental conditions (Fig. S2†). The cells were then centrifuged and the supernatant analysed by UV-vis absorption to determine the amount of COE left in solution (*i.e.* not associated with the pelleted cells). This method is illustrated in Fig. 2 for 10 μM and 20 μM **DSSN+**. Comparing the control spectra of the solutions containing just **DSSN+** in PBS (solid lines) to the spectra of the supernatants resulting from cell staining, one observes that at 10 μM, no discernable **DSSN+** is left in solution, meaning that all COE has associated with the cells. In contrast, at 20 μM a significant absorption is observed indicating that some **DSSN+** remains in the solution and did not associate with the *E. coli*.

**Fig. 3:**
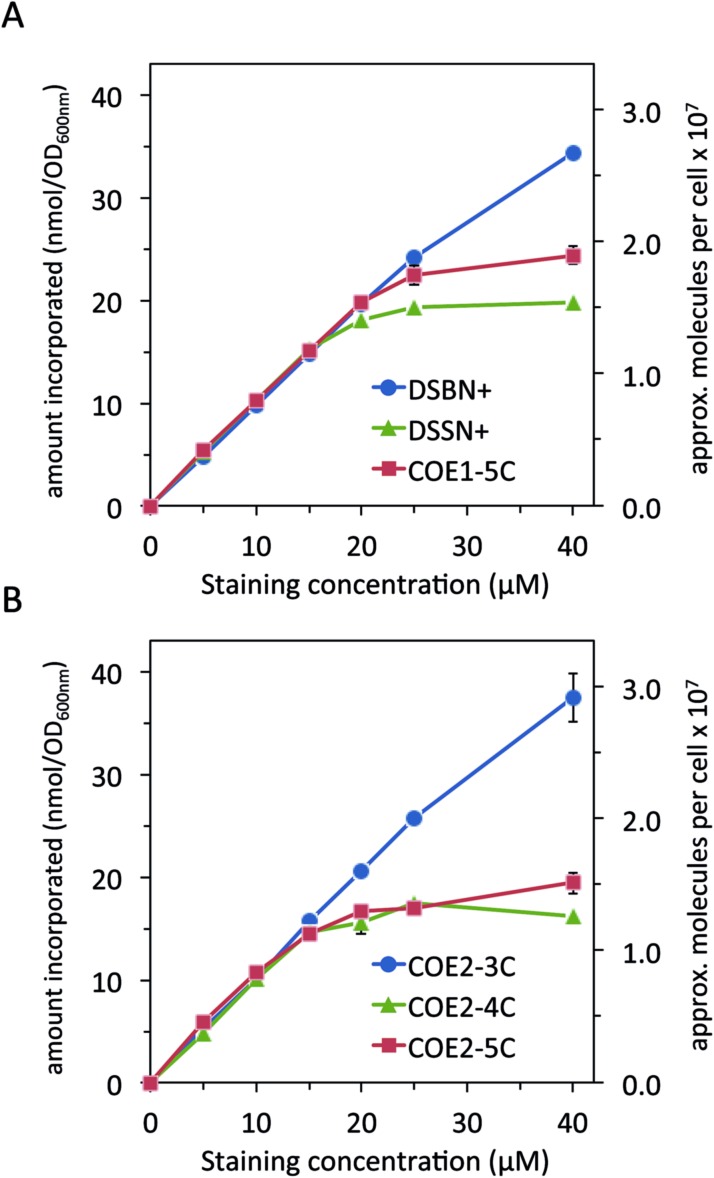
COE associated with *E. coli* cells as a function of staining concentration for (A) COE1 series and (B) COE2 series molecules. The amount of COE associated was calculated by subtracting the absorbance at 420 nm (COE1) or 380 nm (COE2) of the supernatant after centrifugation from that of a control staining solution with no cells. Approximate number of cells assuming 1 OD_600 nm_ = 10^9^ cells per mL.

In subsequent experiments, the amount of COE associated with cells using the UV-vis absorption method was quantified by subtracting the absorbance of the supernatant of stained and centrifuged *E. coli* at a wavelength of 420 nm (COE1 series) or 380 nm (COE2 series) from control samples that did not contain cells. Fig. 3 shows the trends in COE/cell association for the unfluorinated COEs at different staining concentrations normalized to 1 OD_600 nm_ of cells. Interestingly, at concentrations between 1–15 μM for all 6 COEs, 100% association is observed resulting in a linear increase in COE association with increasing staining concentration, reaching ∼15 nmol/OD_600 nm_ associated at 15 μM staining concentration. Looking at the COE1 series in Fig. 3A, at concentrations >15 μM the 4- and 5-RU COEs, **DSSN+** and **COE1-5C**, reach a maximum association of ∼20 ± 0.4 nmol/OD_600 nm_ and ∼25 ± 1.0 nmol/OD_600 nm_ respectively. In contrast, the 3 RU COE, **DSBN+**, does not reach a plateau and attains a maximum association of 34 ± 0.2 nmol/OD_600 nm_ at 40 μM staining concentration. It should be noted that for COEs with minimum inhibitory concentration (MIC) data published (**DSBN+** and **DSSN+**), the MICs (normalized to cell count) required to reduce growth of *E. coli* are 2 orders of magnitude higher than the concentrations used in this study.^13^,^48^ Moreover, it is pointed out in another study, where cytotoxicity tests on *E. coli* with 20 μM of all COE1 series, that no toxicity phenomena is observed in colony forming units (CFUs).^34^

A similar trend is observed for the COE2 series in Fig. 3B with maximum associations of 37 ± 2.4, 18 ± 0.5 20 ± 1.1 nmol/OD_600 nm_ for the 3-, 4- and 5- RU COEs, respectively. When comparing the two series of COEs, the 3- RU COE2 series shows slightly greater maximum association than the 3-RU COE1 series **DSBN+**, suggesting that the structural modification afforded by the alkoxy pendant linkages provide a modest advantage in this respect. However, the comparison between the 4- and 5-RU COEs shows a slightly higher maximum association in the COE1 series than the COE2 series. Interestingly, previous cytotoxicity tests on *E. coli* with 20 μM of all COE2 series showed no toxicity for **COE2-3C**, while **COE2-4C** and **COE2-5C** have demonstrated a ∼30% loss in CFUs than controls.^34^ Regardless of series type, there is a clear dependence of COE association with *E. coli* on molecular length: the amount able to associate with cells for the 4-RU and 5-RU COEs plateaus within the concentration range tested, while the 3-RU COEs do not.

On the secondary *y*-axes in Fig. 3 are the estimated number of COE molecules associated per cell at each staining concentration, with 1 OD_600 nm_ corresponding to a concentration of 10^9^ cells per mL.^49^ With this estimate, it can be seen that maximum COE associations observed in these experiments are greater than 10^7^ molecules per cell for 4- and 5-RU COEs and greater than 2 × 10^7^ for both 3-RU COEs. When comparing these numbers to an estimate of the number of lipids per *E. coli* cell^50^ of ∼2.2 × 10^7^ one can see that the 4- and 5-RU COEs would approach a 1 : 1 lipid : COE ratio in cells and the 3-RU COEs surpass this threshold at the 40 μM staining concentration. As discussed in the Introduction, much evidence has been presented that COEs intercalate into microbial membranes, and up until this point, this has been the only interaction considered. With ratios at or above 1 : 1 lipid : COE per cell, which would be morphologically impossible, it is obvious that not all of the associated COE is intercalating into lipid bilayers. A plausible hypothesis is that some COE is associating with the outside of the *E. coli*, which, with its net negative charge,^51^ is a likely candidate for electrostatic interaction with positively charged molecules.^45^,^52^,^53^

### Zeta potential measurements

**Fig. 4:**
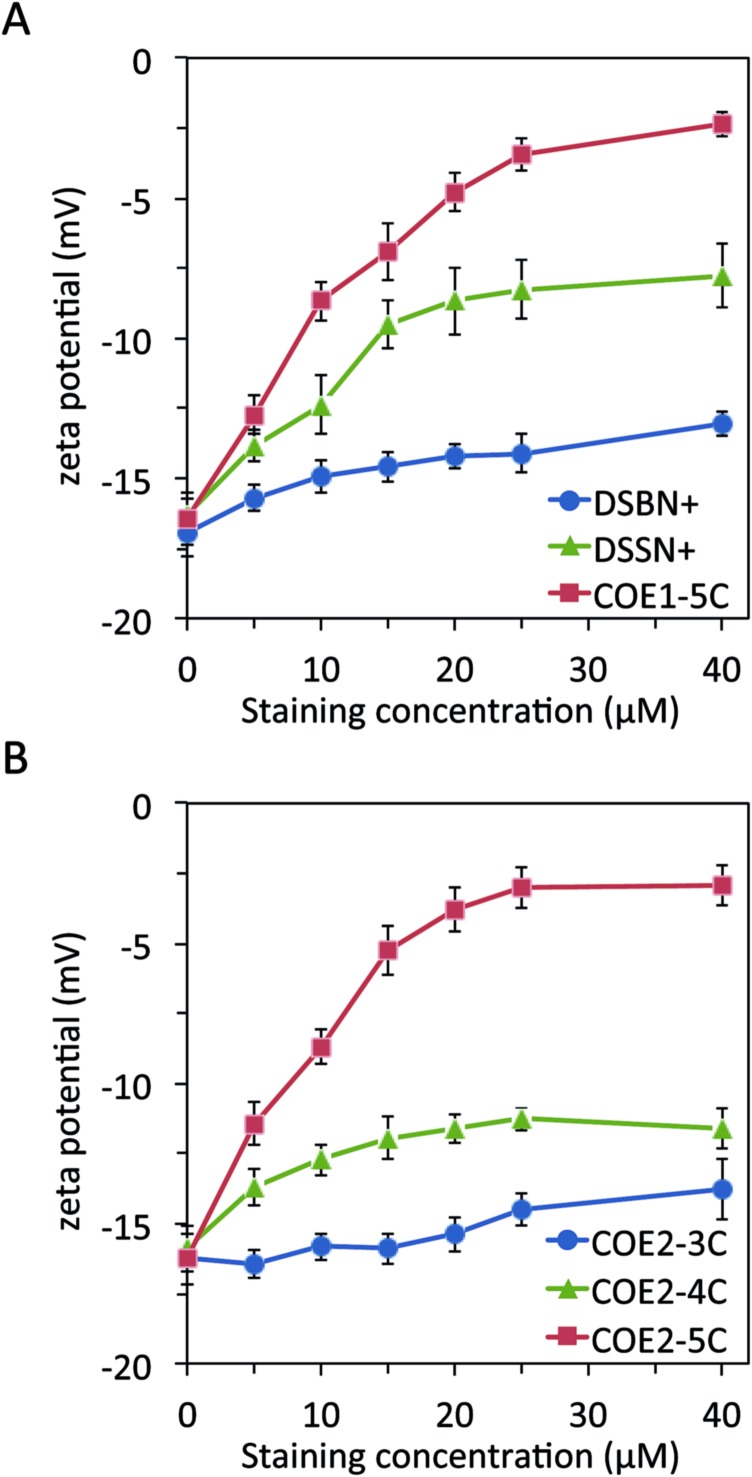
Zeta potential measurements of *E. coli* cells as a function of COE staining concentration for (A) COE1 series and (B) COE2 series. Dashed line represents the zeta potential of unstained *E. coli*.

In order to determine the effect of COE association on cell surface charge, stained *E. coli* cells from the previous experiment were washed and resuspended in PBS buffer for zeta potential measurements,^51^ the results of which are shown in Fig. 4. Unstained cells were found to have an average zeta potential of about –16 mV under these conditions, indicating a net negative charge, as expected.^52^ The cells stained with COE1 series follow a trend of increasing zeta potential to more positive values as the staining concentration increases. Maximum zeta potential values of –13.1 ± 0.4, –7.8 ± 1.1, and –2.4 ± 0.4 mV are reached for the 3-, 4-, and 5-RU COEs, respectively, trending more positive with increasing molecular length. In addition, zeta potential values reflect the association trends observed in Fig. 3A, in that the 4- and 5-RU COEs reach a plateau at a staining concentration around the same concentration that the cell association for these COEs plateaus. Despite having the highest maximum cell association of the COE1 series, the 3-RU COE causes the least change in zeta potential but reflects the association trend in Fig. 3A in that the zeta potential does not appear to plateau in the concentration range tested.

The effect on *E. coli* zeta potential of the COE2 series is shown in Fig. 4B. The COE2 series displays a similar length dependence with maximum zeta potential values for *E. coli* of –13.8 ± 1.1, –11.3 ± 0.6, and –2.9 ± 0.7, observed for **COE2-3C**, **COE2-4C** and **COE2-5C**, respectively. Cells stained by the 3-and 4-RU COE2 molecules display noticeably less positive zeta potential values than their COE1 counterparts but ultimately a similar trend follows in that cells stained by longer COEs result in more positive zeta potential values. Ultimately the change from amine to alkoxy linked pendant groups has only a minor influence on the COE zeta potential effects as a whole.

Rather than observing charge reversal towards high positive values as is seen with cells being coated with positively charged polyelectrolytes,^43^,^44^,^52^ the trend towards charge neutralization in this experiment suggests that not many of the COE positive charges are extending beyond the LPS. COEs are much smaller in size than polyelectrolytes and easily intercalate into lipid membranes and perhaps also ‘interdigitate’ with the oligomeric sugars that form the core of LPS rather than coating the outside cells. In fact, this non-lipid portion of LPS in *E. coli* K12 is estimated to be ∼2.1 nm in length.^54^,^55^ This length is slightly longer than the 3-RU phenylenevinylene core and slightly shorter than the 4-RU conjugated core, which are estimated to be 1.8 nm and 2.4 nm respectively. With the 5-RU core estimated to be around 3 nm, one can begin to rationalize the length scales with the zeta potential results. More specifically, the 4- and 5-RU COEs have a greater chance of spanning the full length or even extending past the outermost LPS units than do the 3-RU COEs, possibly explaining the molecular length dependence of the zeta potential results.

### Fluorinated derivatives

**Fig. 5:**
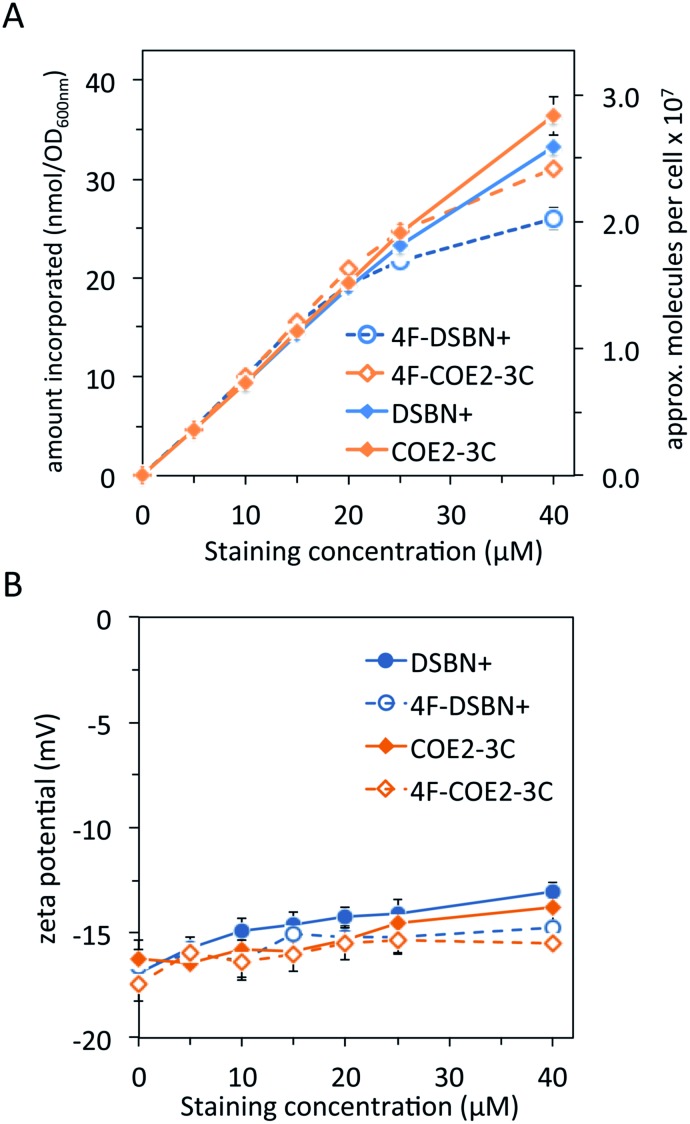
Comparing 3-ring COEs with fluorine substitution (dashed lines, open symbols) and without (solid lines, closed symbols). (A) COE associated with *E. coli* as a function of staining concentration. Approximate number of cells assuming 1 OD_600 nm_ = 10^9^ cells per mL. (B) Zeta potential measurements of stained *E. coli* as a function of COE staining concentration. Black dashed line represents the zeta potential of unstained *E. coli*.

Lastly, cell association and zeta potential experiments were carried out with the fluorine-substituted 3-RU COEs (4FCOEs), the results of which are plotted with the unsubstituted counterparts for comparison and are shown in Fig. 5. Cell association for the 4FCOEs (Fig. 5A) is largely indistinguishable from their unsubstituted counterparts until staining concentrations of ∼25–40 μM, at which point the 4FCOEs associate slightly less. At the highest staining concentration tested (40 μM), there were approximately 2.0 (±0.06) × 10^7^ and 2.4 (±0.02) × 10^7^ molecules associated per cell for **4F-DSBN+** and **4F-COE2-3C**, respectively. These values are 23% and 15% less than for **DSBN+** and **COE2-3C**, respectively. A possible explanation for this deviation at higher staining concentrations is the polar-hydrophobic nature of fluorinated compounds,^56^ making these molecules less likely to aggregate in the lipid membrane due to interactions between the cationic pendant groups and the fluorinated core.^13^ Being less likely to aggregate or pack closely would result in less overall cell association. It is worth noting, however, that aggregation of COEs in a lipid membrane has yet to be experimentally proven.

The zeta potential of *E. coli* stained with the 4FCOEs (Fig. 5B) follows the same trend as the unfluorinated COEs, in that a gradual increase in zeta potential is observed as staining concentration increases. Cells stained with **4F-DSBN+** reach a more positive maximum (–14.8 ± 0.6 mV) than those stained with **4F-COE2-3C** (–15.4 ± 0.6 mV), with both maxima being slightly less positive than the corresponding unfluorinated COEs at –13.1 ± 0.4 mV and –13.8 ± 1.1 mV, respectively. Ultimately, fluorine substitution of the center ring of 3-RU COEs has minimal influence on cell association and zeta potential of stained *E. coli*.

## Conclusions

In conclusion, 8 COEs varying in length and substitutions to the aromatic core have been compared in terms of their association with *E. coli* and their effect on cell zeta potential. Confocal microscopy showed patterns consistent with lipid membrane association for all COEs. At low staining concentrations (<20 μM) nearly 100% of COE in solution associates with cells, leaving none remaining in the supernatant of centrifuged samples. At higher concentrations, 3-RU COEs continue to associate while 4- and 5-RU COEs plateau, reaching a maximum association that cannot be overcome by adding more COE to the staining solution. The 3-RU COEs associate past a 1 : 1 lipid : COE ratio while the 4- and 5-RU COEs approach it, which is morphologically impossible and indicative of cellular association not exclusive to membrane intercalation. Cells stained with COEs generally showed more positive zeta potential values with increasing staining concentration, indicating a neutralization of anionic charges of the LPS by the cationic charges of the COEs. Additionally, more positive zeta potential values were observed for longer COEs suggesting that they are able to extend beyond the negatively charged molecular constructs of the *E. coli* LPS. The other structural variations presented here, namely amine *vs.* alkoxy pendant linkages and fluorination of the aromatic core, proved less important than molecular length, as they had minimal effects on cell association and zeta potential, when compared to analogues with the same number of repeat units. These changes alter the photophysical properties of the molecules and thus increase the number of COEs available for applications in bioimaging^19^,^20^,^57^–^59^ and optoelectronics.^60^,^61^ Most importantly, that the zeta potential of bacteria can be tuned by COE length and concentration has implications for technologies such as microbial electronics, wastewater treatment, and others that rely on bacterial aggregation, adhesion and biofilm formation.^62^–^68^

## Experimental

### Materials

All materials were used as received and purchased from Sigma-Aldrich or Fisher Scientific unless otherwise noted.

### Cell culture

*Escherichia coli* K-12 (ATCC 10798) was grown aerobically in Luria Broth (10 g L^–1^ bacto tryptone, 5 g L^–1^ yeast extract, 10 g L^–1^ NaCl) overnight at 37 °C with shaking.

### Cell staining for microscopy

Before staining, *E. coli* was rinsed twice from the growth medium with phosphate buffered saline (PBS) containing the following: 45.7 mM NaCl, 0.9 mM KCl, 3.3 mM Na_2_HPO_4_ and 0.6 mM KH_2_PO_4_ at pH 7.4. 0.5 mL of OD_600_ = 0.9 cells were stained with 10 μM COE for 1 hour in the dark at room temperature before rinsing twice. Samples were then resuspended in 100 μL of PBS and 5 μL were dropped onto a clean glass slide and a cover slip placed on top. Cover slips were sealed with clear nail polish and all samples were imaged within 2 hours.

### Confocal microscopy

All images were obtained *via* laser scanning confocal microscopy using an Olympus FluoView 1000S spectral scanning microscope equipped with a 60 × 1.30 silicon oil immersion lens. A 405 nm laser was used as the excitation source. For the COE1 series, emission was collected from 480 nm–580 nm. For the COE2 series, emission was collected from 410 nm–510 nm. All images were processed using ImageJ.

### COE cell association experiments

*E. coli* cells at OD_600 nm_ = 1.0 were stained in clear 96-well plates (BD Biosciences, San Jose, CA) at 20 °C for 1 hour in the dark with shaking. Total volume of each sample was 200 μL and samples were measured in triplicate. After centrifugation of the plate (3500 rpm, 4 minutes), 100 μL of supernatant was transferred to a clean well for UV-vis absorption with a Tecan M220 Infinite Pro plate reader (Tecan, Männedorf, Switzerland). Absorbance was measured at 420 nm for COE1 series and 380 nm for COE2 series molecules. Control samples with no cells were treated the same and their absorbance values represented the total COE from which the supernatant values were subtracted to give the amount associated with cells.

### Zeta potential measurements

Stained, twice-rinsed cells were resuspended in PBS to their original OD_600 nm_ = 1.0. 100 μL of each sample was diluted into 900 μL PBS for zeta potential measurements on a Malvern Zetasizer Nano ZS (Malvern Instruments, Malvern, U.K.) at 20 °C. Data points given are an average of 4 biological replicates with 3 measurements each.

## Supplementary Material

Supplementary information
