# Supplementary material for: Tuning cell surface charge in E. coli with conjugated oligoelectrolytes
Source: Chem Sci. 2015 Dec 3;7(3):2023–9. doi: 10.1039/c5sc03046c (PMC5968544; doi:10.1039/c5sc03046c)
Supplement: Supplementary file 1 [file SC-007-C5SC03046C-s001.pdf]

Electronic Supplementary Material (ESI) for Chemical Science.
This journal is © The Royal Society of Chemistry 2015

Supporting Information

Tuning Cell Surface Charge in *E. Coli* with Conjugated Oligoelectrolytes

Chelsea Catania, Alexander W. Thomas and Guillermo C. Bazan*

Cell culture

Escherichia coli K-12 (ATCC 10798) was grown aerobically in Luria Broth (10 g L^{-1} bacto tryptone, 5 g L^{-1} yeast extract, 10 g L^{-1} NaCl) overnight at 37°C with shaking.

Cell staining for microscopy

Before staining, *E. coli* was rinsed twice from the growth medium with phosphate buffered saline (PBS) containing the following: 45.7 mM NaCl, 0.9 mM KCl, 3.3 mM Na_2HPO_4 and 0.6 mM KH_2PO_4 at pH 7.4. 0.5 mL of OD_{600} = 0.9 cells were stained with 10 µM COE for 1 hour in the dark at room temperature before rinsing twice. Samples were then resuspended in 100 µL of PBS and 5 µL were dropped onto a clean glass slide and a cover slip placed on top. Cover slips were sealed with clear nail polish and all samples were imaged within 2 hours.

Confocal microscopy

All images were obtained via laser scanning confocal microscopy using an Olympus FluoView 1000S spectral scanning microscope equipped with a 60 x 1.30 silicon oil immersion lens. A 405 nm laser was used as the excitation source. For the COE1 series, emission was collected from 480 nm – 580 nm. For the COE2 series, emission was collected from 410 nm – 510 nm. All images were processed using ImageJ.[1]

COE cell association experiments

E. coli cells at OD_{600} = 1.0 were stained in clear 96-well plates (BD Biosciences, San Jose, CA) at 20 °C for 1 hour in the dark with shaking. Total volume of each sample was 200 µL. After centrifugation of the plate (3500 rpm, 4 minutes), 100 µL of supernatant was transferred to a clean well for UV-Vis absorption with a Tecan M220 Infinite Pro plate reader (Tecan, Männedorf, Switzerland). Absorbance was measured at 420 nm for COE1 series and 380 nm for COE2 series molecules. Control samples with no cells were treated the same and their absorbance values represented the total COE from which the supernatant values were subtracted to give the amount associated with cells. All conditions and controls were measured in triplicate.

Zeta potential measurements

Stained, twice-rinsed cells were resuspended in PBS to an OD_{600nm} = 1.0. 100 µL of each sample was diluted into 1 mL PBS for zeta potential measurements on a Malvern Zetasizer Nano ZS (Malvern Instruments, Malvern, U.K.) at 20 °C. Data points given are an average of 4 biological samples with 3 measurements for each sample.

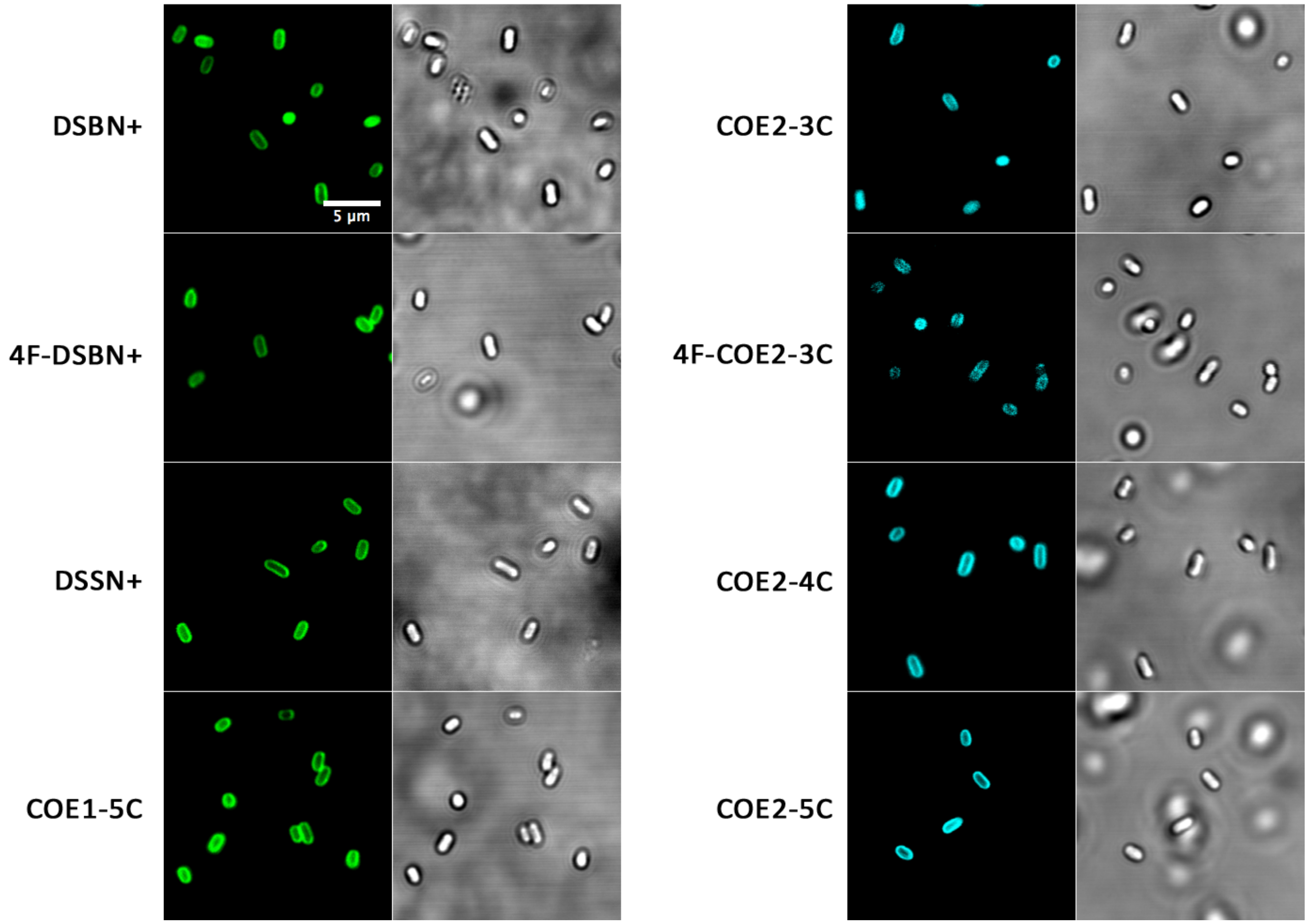

Figure S1. Laser scanning confocal micrographs and accompanying brightfield images of *E. coli* stained with 10 µM COE in PBS for 1 hour. Excitation wavelength was 405 nm for all images. 5 µm scale bar is the same for all images.

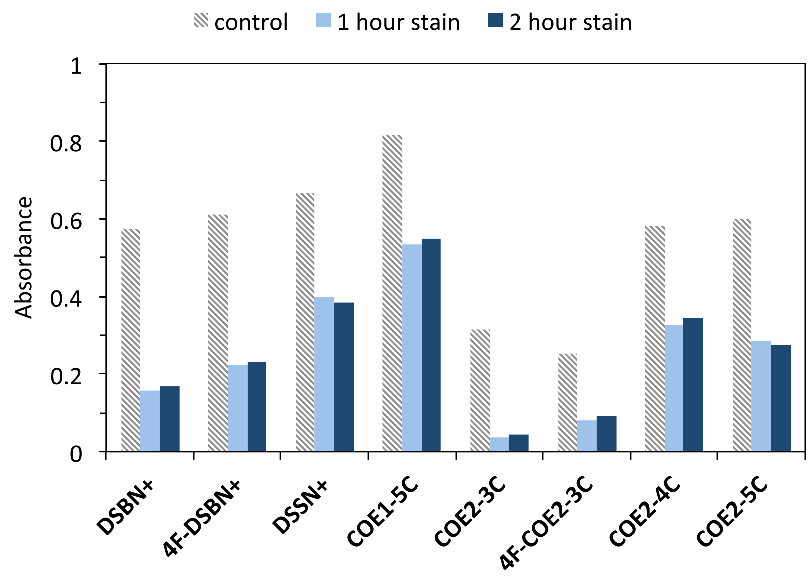

Figure S2. In order to demonstrate that the system had reached equilibrium after 1 hour, the supernatant of *E. coli* stained with 40 µM COE for 1 hour (light blue) and 2 hours (dark blue) were analysed by UV-vs absorption at 420 nm (COE1 series) and 380 nm (COE2 series). Note that a lower absorbance in the supernatant indicates less COE left in solution and more associated with cells. Shown in grey are 40 µM COE solutions in PBS (*i.e*. the amount of COE in solution with no cells present).

References

1 W. S. Rasaband, ImageJ: U. S. National Institutes of Health, Bethesda, Maryland, USA, http://imagej.nih.gov/ij/, 1997-2012.
